# The prognostic value of concomitant assessment of NT-proCNP, C-reactive protein, procalcitonin and inflammatory cytokines in septic patients

**DOI:** 10.1186/cc13944

**Published:** 2014-06-25

**Authors:** Ryszard Tomasiuk, Malgorzata Mikaszewska-Sokolewicz, Stanislaw Szlufik, Piotr Rzepecki, Tomasz Lazowski

**Affiliations:** 1 Department of Laboratory Diagnostics, Mazovian Brodno Hospital in Warsaw, Warsaw 03-242, Poland; 2 Department of Anesthesiology and Intensive Care, Medical University of Warsaw, Warsaw 02-091, Poland; 3 Department of Neurology, Faculty of Heath Science, Medical University of Warsaw, Warsaw 02-091, Poland

## Findings

The mortality of patients with sepsis in ICUs has been decreasing since 1991 [1]. Nevertheless, there are still conflicting data regarding potential biomarkers of mortality rate among critically ill patients and sepsis development. N-terminal pro C-type natriuretic peptide (NT-proCNP) is one of the markers whose usefulness is still uncertain. Some authors have shown the possible role of NT-proCNP in the prognosis of critically patients with sepsis [2] and in polytrauma patients without brain injury who developed sepsis [3], but Gouel-Chéron and colleagues [4] showed no correlation between the serum level of NT-proCNP and development of sepsis.

Our study aimed to explore the serum level of NT-proCNP and its potential triggers (TNF-α, IL-6, IL-8, IL-10, IL-12) and to correlate them with serum levels of procalcitonin (PCT) and C-reactive protein (CRP) as reviewed by Uzzan and colleagues [5].

Samples were obtained from 90 critically ill adult patients during the first day in the ICU. The patients were divided in two groups: patients who died of sepsis in the ICU (43 patients), and ICU survivors (47 individuals). All patients were assessed with the use of Acute Physiology and Chronic Health Evaluation II score. ELISA kits were used for quantitative determination of human NT-proCNP. Cytokines were measured with the Fluorokine® MAP cytokine multiplex kit and the Luminex® 100 Platform. Statistical evaluation included Mann–Whitney U test and Spearman correlation coefficient.

The median serum level of NT-proCNP was significantly higher in non-survivors than in survivors: 7.1 (interquartile range (IQR) 3.7 to 18.1) pmol/L versus 4.5 (IQR 2.3 to 7.2) pmol/L, *P* < 0.05. Likewise, serum CRP levels were higher in non-survivors than in survivors: 132.5 (IQR 77.6 to 180.8) mg/L versus 93.8 (IQR 50.1 to 162.4 ) mg/L, *P* < 0.05. The differences in median PCT, IL-6, IL-8, IL-10, IL-12 and TNF- α levels did not reach statistical significance (Table 1).

**Table 1:** Median serum levels of NT-proCNP, C-reactive protein, procalcitonin and inflammatory cytokines in non-survivors and survivors

	**Median (interquartiles)**	
**Parameter**	**Non-survivors**	**Survivors**
NT-proCNP* (pmol/L)	7.1 (3.7 to 18.1)	4.5 (2.3 to 7.2)
PCT (ng/ml)	16.2 (2.5 to 36.2)	11.2 (2.1 to 38.4)
CRP* (mg/L)	132.5 (77.6 to 180.8)	93.8 (50.1 to 162.4)
IL-6 (pg/mL)	8.6 (4.0 to 47.6)	12.9 (5.5 to 57.5)
IL-8 (pg/mL)	167.7 (34.9 to 446.4)	175.2 (50.2 to 518.7)
IL-10 (pg/mL)	21.0 (9.0 to 57.0)	28.4 (12.0 to 66.0)
IL-12 (pg/mL)	3.5 (2.4 to 4.7)	3.1 (2.0 to 4.8)
TNF-α (pg/mL)	4.5 (1.6 to 6.2)	4.7 (1.6 to 6.0)

*P < 0.05. CRP, C-reactive protein; IL, interleukin; NT-proCNP, N-terminal pro C-type natriuretic peptide; PCT, procalcitonin; TNF, tumor necrosis factor.

Our study is in agreement with the studies by Koch and colleagues [2] and Bahrami and colleagues [3], supporting the possibly important role of NT-proCNP as a prognostic biomarker in sepsis. Inflammatory cytokines (like IL-1 and TNF-α) are known to trigger the release of CNP from endothelial cells but, interestingly, this was not confirmed in our study (median levels of IL-1, TNF-α, IL-6, and IL-10 were not statistically correlated with survival). In our study PCT was not a good prognostic factor either (possibly due to the small cohort of patients).

Determination of CRP and NT-proCNP can potentially determine the risk of death. Outcome prediction based on these biomarkers may have sound clinical implications. This requires further study in larger patient populations.

## Abbreviations

CRP: C-reactive protein; ELISA: Enzyme-linked immunosorbent assay; IL: Interleukin; IQR: Interquartile range; NT-proCNP: N- terminal pro C-type natriuretic peptide; PCT: Procalcitonin; TNF: Tumor necrosis factor.

## Competing interests

The authors declare that they have no competing interests.
